# Rapamycin-Loaded Polymeric Nanoparticles as an Advanced Formulation for Macrophage Targeting in Atherosclerosis

**DOI:** 10.3390/pharmaceutics13040503

**Published:** 2021-04-07

**Authors:** Emanuela Fabiola Craparo, Marta Cabibbo, Alice Conigliaro, Maria Magdalena Barreca, Teresa Musumeci, Gaetano Giammona, Gennara Cavallaro

**Affiliations:** 1 Department of Biological, Chemical and Pharmaceutical Science and Technologies (STEBICEF), University of Palermo, Via Archirafi 32, 90123 Palermo, Italy; marta.cabibbo@unipa.it (M.C.); gaetano.giammona@unipa.it (G.G.); 2 Department of BioMedicine, Neuroscience and Advanced Diagnostics (Bi.N.D), University of Palermo, Via Divisi 83, 90133 Palermo, Italy; alice.conigliaro@unipa.it (A.C.); mariamagdalena.barreca@unipa.it (M.M.B.); 3 Laboratory of Drug Delivery Technology, Department of Drug Sciences, University of Catania, Via Santa Sofia 64, 95125 Catania, Italy; teresa.musumeci@unict.it

**Keywords:** rapamycin (Rapa), polymeric nanoparticles, macrophage targeting, KOdia-PC, atherosclerosis

## Abstract

Recently, rapamycin (Rapa) represents a potential drug treatment to induce regression of atherosclerotic plaques; however, its use requires site-specific accumulation in the vessels involved in the formation of the plaques to avoid the systemic effects resulting from its indiscriminate biodistribution. In this work, a stable pharmaceutical formulation for Rapa was realized as a dried powder to be dispersed extemporaneously before administration. The latter was constituted by mannitol (Man) as an excipient and a Rapa-loaded polymeric nanoparticle carrier. These nanoparticles were obtained by nanoprecipitation and using as a starting polymeric material a polycaprolactone (PCL)/α,β-poly(*N*-2-hydroxyethyl)-dl-aspartamide (PHEA) graft copolymer. To obtain nanoparticles targeted to macrophages, an oxidized phospholipid with a high affinity for the CD36 receptor of macrophages, the 1-(palmitoyl)-2-(5-keto-6-octene-dioyl) phosphatidylcholine (KOdia-PC), was added to the starting organic phase. The chemical–physical and technological characterization of the obtained nanoparticles demonstrated that: both the drug loading (DL%) and the entrapment efficiency (EE%) entrapped drug are high; the entrapped drug is in the amorphous state, protected from degradation and slowly released from the polymeric matrix; and the KOdia-PC is on the nanoparticle surface (KP-Nano). The biological characterization demonstrated that both systems are quickly internalized by macrophages while maintaining the activity of the drug. In vitro studies demonstrated that the effect of KP-Nano Rapa-loaded, in reducing the amount of the Phospo-Ser757-ULK1 protein through the inhibition of the mammalian target of rapamycin (mTOR), is comparable to that of the free drug.

## 1. Introduction

Cardiovascular diseases caused by atherosclerosis represent a serious problem for society, both in terms of lost lives and the economic investment linked to their management [1,2]. The possibility of managing them through innovative, minimally invasive therapies that also aim at early diagnosis represents the challenge of current research [3].

Since atherosclerosis is triggered by a series of processes that result from the formation of atherosclerotic plaques in the blood vessels, the possibility of highlighting them as soon as possible and treating them to block their growth represents a valid possibility of reducing its consequences on human health. The pathogenetic mechanisms that determine the formation of atherosclerotic plaques in blood vessels are manifold and range from altered lipid metabolism to the involvement of an abnormal immune-inflammatory response [4]. The current pharmacological treatment allows for reducing the risks of acute coronary events such as thrombosis, but it does not lead to a regression of the disease nor normalize the aberrant activity of the cells involved in the process. Therefore, current research aims at the discovery of new therapeutic possibilities that reduce the risk of the onset of the pathology and slow down its progression, by acting on specific anti-inflammatory and immunomodulatory mechanisms of the involved cells.

Recently, rapamycin (Rapa), has been proposed as a potential drug treatment for the management of atherosclerosis [5,6]. Currently, Rapa is administered in clinics as an immunosuppressant to avoid allograft rejection in kidney transplantation. Due to its cellular functions and molecular mechanisms, it shows high potential in the treatment of other various human pathologies, showing immunosuppressive, anti-proliferative, antiangiogenic, antifungal, anti-restenosis, and anti-inflammatory properties, but whose real applicability is severely limited by formulation problems and poor bioavailability [7,8]. Moreover, severe effects are associated with the inhibition of (mammalian target of rapamycin) (mTOR)-regulated pathways, such as immunosuppression, dyslipidemia, and hyperglycemia due to nonspecific biodistribution after the oral administration of Rapa [5,6].

Just by inhibiting the protein kinase mTOR, Rapa could show multiple protective effects in the cells most involved in the progression of atherosclerosis, such as macrophages, endothelial cells, and the smooth muscle cells of altered vessels. In support of this, it was recently reported in the literature that Rapa can inhibit inflammatory immune response and growth-factor-driven smooth muscle cell (SMC) proliferation, prevent monocyte recruitment and lipid accumulation in macrophages and SMC, and stimulate autophagy [9].

To reduce the risk of serious effects due to non-specific biodistribution, site-specific accumulation at the level of the vessel involved in the formation of a plaque would be a possible strategy to pave the way for the use of Rapa in the treatment of atherosclerosis [7]. In the literature, there are already numerous attempts to formulate Rapa through innovative carriers such as liposomes, polymeric nanoparticles, and other formulations, with encouraging results both for the diagnosis and for the treatment of numerous pathologies [7,10,11]. The advantages could be either the improvement of the chemical–physical properties of the drug itself or optimization of bioavailability, both thanks to the modification of the drug release profile and the carrier site-specific directing effect [12,13]. Research efforts to optimize and make real the use of innovative carriers also for the detection and treatment of atherosclerosis are already well-documented in the literature [3,14,15,16,17].

Recent encouraging results concerning Rapa have already been obtained with innovative biomimetic polymeric carriers [18,19,20,21]. Macrophages are the cells that play a key role in the formation, progression, and destabilization of atherosclerotic plaques, by transforming into foam cells and constituting the majority of apoptotic cells that influence the size and vulnerability of the plaque. Therefore, the development of polymeric carriers targeting these cells would seem to represent a winning strategy for the treatment of atherosclerosis [17,22,23,24]. By exploiting the fact that macrophages accumulate cholesterol deriving from native Low Density Lipoproteins (LDL) and oxidized low-density lipoproteins (oxLDL) through a mechanism mediated by numerous scavenger receptors such as the CD36 receptor, oxidized phospholipids appear to be the most plausible ligands for oxLDL binding to the receptor.

In this paper, a powder formulation containing Rapa, characterized to propose them for parenteral administration for the treatment of atherosclerosis, was developed and optimized. In particular, this formulation was obtained by drying an aqueous dispersion containing mannitol (Man) and Rapa-loaded polymeric nanoparticles. The latter particles consisted of a biocompatible polymer obtained starting from the polycaprolactone (PCL) /α,β-poly(*N*-2-hydroxyethyl)-dl-aspartamide (PHEA) graft copolymer, and of an oxidized phospholipid, the 1-(palmitoyl)-2-(5-keto-6-octene-dioyl)phosphatidylcholine (KOdia-PC), chosen because it shows the highest affinity for the CD36 receptor of macrophages, to obtain a system capable of accumulating selectively in the atherosclerotic plaques [25]. The obtained particles were characterized in terms of KOdia-PC and Rapa content, thermal analysis, mean size before and after drying by freeze-drying or spray-drying, drug stability, and release after entrapment into the particles. Moreover, the targeting effect of KOdia-PC present on the nanoparticle surface and drug activity, free or loaded into nanoparticles targeted to macrophages, was also evaluated by biological assays.

## 2. Materials and Methods

### 2.1. Materials

Anhydrous *N,N’*-dimethylformamide (a-DMF), Poly-ε-caprolactone (PCL, $\bar{M}$ = 10,000–18,000 Da), succinic anhydride (SA), dimethylaminopyridine (DMAP), 1,1′-carbonyldiimidazole (CDI), mannitol (Man), Poly(ethylene glycols) standards, dimethylacetamide (DMA), were purchased from Sigma-Aldrich (Milan, Italy). Diethylamine (DEA), tetrahydrofuran (THF), diethyl ether, dichloromethane, methanol, acetone, Dulbecco’s phosphate buffer saline (DPBS), foetal bovine serum (FBS), were obtained from Fluka (Milan, Italy). 1-(palmitoyl)-2-(5-keto-6-octene-dioyl) phosphatidylcholine (KOdia-PC) was purchased from Cabru (Monza, Italy). Rapamycin (Rapa) was obtained from Accel Pharmatech (NJ, USA). All reagents were of analytic grade, unless otherwise stated.

α,β-poly(*N*-2-hydroxyethyl)-dl-aspartamide (PHEA) and PHEA-g-RhB were properly synthesized by following procedures already reported in the literature [26,27].

^1^H-NMR spectra were registered by a Bruker Avance II-300 spectrometer, working at 300 MHz (Bruker, Milan, Italy).

Size exclusion chromatography (SEC) analysis was done by using a Waters system (Waters, Mildford, MA, USA), equipped with two Phenogel columns (particle size of 5 μm, with pores size of 10^3^ Å and 10^4^ Å) from Phenomenex (Bologna, Italy), and a refractometer as detector, at 50 °C. 0.01 M LiBr DMF solution was used as eluent phase at a flow of 0.8 mL/min, while PEG (with an average molecular weight in the range of 145,000–1500 Da) were used as standards. The copolymer was dispersed in the LiBr solution used as eluent, filtered with a filter (0.2 μm as pore size) and analyzed in triplicate.

PHEA ^1^H-NMR (300 MHz, D_2_O, 25 °C, TMS): δ 2.71 (m, 2H_PHEA_, –COCHCH_2_CONH–), δ 3.24 (m, 2H_PHEA_, –NHCH_2_CH_2_O–), δ 3.55 (m, 2H_PHEA_, –NHCH_2_CH_2_OH), δ 4.59 [m, 1H_PHEA_, –NHCH(CO)CH_2_–]. The weight-average molecular weight (${\bar{M}}_{w}$) of PHEA used in this study, determined by size exclusion chromatography (SEC) analysis, was 53.6 kDa (${\bar{M}}_{w}/{\bar{M}}_{n}$ = 1.2).

PHEA-g-RhB ^1^H-NMR (300 MHz, D_2_O, 25 °C, TMS): δ 1.15 (12H_RhB_ CH_3_CH_2_–); δ 2.71 (2H_PHEA_ –COCHCH_2_CONH–); δ 3.29 (2H_PHEA_ –NHCH_2_CH_2_O–); δ 3.58 (2H_PHEA_ –NHCH_2_CH_2_O–); δ 4.65 (1H_PHEA_ –NHCH(CO)CH_2_–); δ 8.00-8.50 (10H_RhB_ H–Ar). The ${\bar{M}}_{w}$ of PHEA-g-RhB used in this study was 52.5 Da (${\bar{M}}_{w}/{\bar{M}}_{n}$ = 1.6). The degree of derivatization in RhB (DD_RhB_), determined from the ^1^H-NMR spectra, as reported elsewhere, was equal to 0.6 ± 0.05 mol% [27].

### 2.2. Synthesis and Characterization of Poly-ε-Caprolactone-Succinate (PCL-SUCC)

The functionalization of PCL with succinic acid was done accordingly to the procedure recently reported in the literature [28]. Briefly, a proper amount of PCL was dispersed in a-DMF (133.4 mg/mL), and to the obtained solution dimethylamino pyridine (DMAP) and succinic anhydride (SA) were subsequently added. The amounts of DMAP and SA were properly defined by R_1_ = 40 and R_2_ = 1.2, where R_1_ is the molar ratio between SA and PCL and R_2_ the molar ratio between DMAP and PCL. The obtained solution was placed in a heating oil plate at 60 °C. After 24 h, the mixture was precipitated dropwise in cold diethyl ether and vacuum-dried, and the product was purified by washing several times with twice-distilled water. After that, the obtained product was dissolved in acetone, dialyzed against twice-distilled water (MWCO 12–14 kDa), and the obtained aqueous suspension freeze-dried by using a Labconco freeze-dryer (Kansas City, MO, USA).

The obtained white solid product, PCL-SUCC, was characterized in terms of carboxylation degree by titration in THF with a KOH methanol solution (35.6 mM), using a THF solution of thymol blue (5 mg/mL) as an indicator.

### 2.3. Synthesis and Characterization of PHEA-g-RhB-g-SUCC-PCL

The grafting reaction of PCL-SUCC on PHEA-g-RhB was carried out via CDI (as condensing agent) to obtain the PHEA-g-RhB-g-SUCC-PCL graft copolymer [28]. PCL-SUCC was dispersed in a-DMF (66.5 mg/mL), and then CDI was added in a proper amount calculated according to R_3_ = 3, that is, the mole ratio between CDI and PCL-SUCC. The resultant dispersion was left at 40 °C for 5 h. Meanwhile, PHEA-g-RhB was dispersed in a-DMF (33 mg/mL) at 40 °C and DEA was added as a catalyst, in a proper amount to obtain R_4_ = 0.3, that is, the molar ratio between DEA and those of repeating units (RUs) of PHEA-g-RhB. The latter dispersion was added dropwise to the CDI-activated PCL-SUCC dispersion, according to R_5_ = 0.06, that is, the molar ratio between PCL-SUCC and PHEA-g-RhB RUs. The mixture reaction was left under continuous stirring at 40 °C. After 44 h, the mixture was added dropwise in diethyl ether, and the obtained precipitate was recovered by centrifugation (at 4 °C for 15 min, at 9800 rpm) with a Coulter Allegra X-22R refrigerated centrifuge (Beckman, Milan, Italy), and purified by washing several times with a diethyl ether: dichloromethane mixture (4:1 *v/v*). The recovered solid was dried under reduced pressure, dissolved in DMA, dialyzed against water (MWCO 12–14 kDa), and freeze-dried.

^1^H-NMR (300 MHz, [D7].DMF, 25 °C, TMS): δ 1.5 and 2.1 (m, 6H_PCL_ –[O(O)CCH_2_(CH_2_)_3_CH_2_]_122_–); δ 2.5 (2d, 2H_PCL_ –[O(O)CCH_2_(CH_2_)_3_CH_2_]_122_–); δ 2.8 (m, 2H_PHEA_ –C(O)CHCH_2_C(O)NH–); δ 3.2 (t, 2H_PHEA_ –NHCH_2_CH_2_O-); δ 3.50 (t, 2H_PHEA_ –NHCH_2_CH_2_O–); δ 4.3 (t, 2H_PCL_ –[O(O)CCH_2_(CH_2_)3CH_2_]_122_–), and δ 5.0 (m, 1H_PHEA_ –NHCH(CO)CH_2_–).

### 2.4. Nano-Precipitation

Nanoparticles (sample Nano) were produced by adding dropwise, by a burette, a PHEA-g-RhB-g-SUCC-PCL graft copolymer dispersion (1.5% *w/v* in DMA) to bidistilled water (1:10 *v/v*), at a flow rate equal to 1 mL/min. The obtained dispersion was stirred for 2 h, then purified by dialysis against twice-distilled water using a dialysis tube with a MWCO of 12–14 kDa. The content of the dialysis tube was then recovered and freeze-dried, and the solid product stored at −20 °C for further characterization. 

To produce targeted nanoparticles (sample KP-Nano), KOdia-PC was added to the organic phase at a copolymer:ligand ratio of 60:1, which was then added dropwise to the aqueous water. To produce drug-loaded nanoparticles, the same procedure was followed by adding the Rapa into the DMA organic phase (0.32% *w/v*) containing the KOdia-PC and/or the copolymer alone, obtaining, respectively, the samples Rapa-KP-Nano and Rapa-Nano.

### 2.5. Nanoparticle Drying

Drying of the nanoparticle dispersion, containing 1 wt% Man, was carried out by freeze-drying using a Modulyo freeze-dryer (Labconco Corporation, Kansas City, MO, USA), and by spray-drying using a Büchi Nano Spray Dryer B-90 (Büchi, Milan, Italy).

The morphology of spray-dried particles was evaluated using a Phenom™ ProX Desktop SEM microscope.

### 2.6. Nanoparticle Characterization

#### 2.6.1. Size and ζ Potential Measurements

Empty and drug-loaded Nano were characterized in terms of hydrodynamic diameter (Z-Average), PDI, and ζ potential, using a Malvern Zetasizer Nano ZSP instrument (Malvern Instrument, Malvern, UK) with a He-Ne laser at λ = 632.8 nm and at a fixed scattering angle of 175°. Each sample was dispersed in ultrapure water and analyzed at 25 °C, repeating the measurement in triplicate. The intensity-average hydrodynamic diameter and polydispersity index (PDI) were obtained by a cumulant analysis of the correlation function.

The Zeta potential (mV) was calculated from the electrophoretic mobility using the Smoluchowsky relationship and assuming that Ka >> 1 (where K and a are the Debye-Hückel parameters and particle radius, respectively). Each measurement was repeated in triplicate.

#### 2.6.2. X-ray Photoelectron Spectroscopy (XPS) Analysis

XPS spectra were recorded on each sample by using a PHI 5000 VersaProbe II (ULVAC-PHI, Inc., Chigasaki, Japan) and monochromatic Al-Kα radiation (hν = 1486.6 eV) from an X-ray source operating at a spot size of 200 μm, a power of 50 W, and an acceleration voltage of 15 kV.

#### 2.6.3. Kodia-PC Quantification

The quantification of Kodia-PC was carried out by a colorimetric method developed for the quantification of phospholipids in biological samples, which is based on the formation of a complex between phospholipid and ammonium ferrothiocyanate, quantifiable by UV analysis [29,30]. Briefly, the assay was carried out in glass vials containing equal volumes of ammonium ferroisothiocyanate solution, and chloroform, where an exactly weighted amount of KP-Nano was dispersed. The system was shaken vigorously for 15 min, then the lower chlorinated solvent layer was separated and clarified with anhydrous sodium sulfate, if necessary. Then it was placed into a quartz cuvette and the absorbance was measured at 488 nm by a RF-5301PC spectrofluorometer (Shimadzu, Italy). Incubations were performed in triplicate and each experiment was repeated at least three times. A calibration curve was obtained by analyzing the Kodia-PC dispersion in chloroform at concentrations ranging between 0.01–0.05 mg/mL (y = 10.068x, R^2^ = 0.998).

#### 2.6.4. Differential Scanning Calorimetry (DSC) Analysis

Thermotropic evaluations were carried out with a Mettler Toledo DSC 1 STARe system equipped with a PolyScience temperature controller (PolyScience Niles, Illinois, USA). The sensitivity was automatically chosen as the maximum possible through the calorimetric system, and the reference was an empty pan (signal time constant 18 s; digital resolution of the measurement signal < 0.04 µW, calorimetric resolution 0.12, and sensitivity 11 both determined through the TAWN test; the sampling rate 50 values/second). Calibration was carried out using indium as described in the procedure of the DSC 1 Mettler TA STARe instrument. Raw materials and nanoparticles were sealed in an aluminum pan and submitted to DSC analysis to determine the thermotropic parameters of the samples. Each sample was submitted to heating and cooling cycles in the temperature range of 10–200 °C at a scanning rate of 5 °C/min (heating) and a scanning rate of 10 °C/min (cooling). The transition temperature was calculated from peak areas with the Mettler STARe Evaluation software system (version 16.20).

### 2.7. Rapa Entrapment

#### 2.7.1. Determination of Drug Loading (DL%)

The determination of drug loading (DL%) as the Rapa amount was loaded into nanoparticle samples (KP-Nano or Nano) was done by HPLC. A Waters Breeze System Liquid Chromatograph system equipped with an autosampler (40 μL injection volume) and a UV−vis HPLC detector were used. The chosen column was a Luna^®^ C18 (C18(2) 100A 250 × 4.6 mm, from Phenomenex); a mixture of methanol and water 70:30 *v/v* was used as the mobile phase at a flow rate of 1 mL/min, at 25 °C. The chosen wavelength for the detection was 277 nm. A calibration curve was previously obtained by plotting areas versus standard solution concentrations of Rapa in methanol in the range of 0.02–0.001 mg/mL (y = 136513x, R² = 0,9994), retention time = 14 min.

Each nanoparticle sample was properly dissolved in DMA and methanol to extract the Rapa (1:9 *v/v*, 2.5 mg/mL). The obtained dispersion was filtered (with filters at a 0.45 μm pore size) and the obtained solution was analyzed by HPLC. Each obtained peak area at 14 min was compared with the calibration curve. DL% results were expressed as the weight percent ratio between the total loaded Rapa and the dried system (Rapa-loaded KP-Nano or Rapa-loaded Nano). Entrapment efficacy (EE%) was expressed as the weight percent ratio between the amount of Rapa entrapped into the particles and the theoretical one.

#### 2.7.2. Drug Stability

The stability of Rapa was evaluated by determining the amount of intact drug over time after incubation in proper conditions. In particular, a proper amount of drug was weighed, dissolved in methanol, and 50 µL (0.09 mg of drug) were added to 30 mL of a mixture between DPBS:fetal bovine serum (FBS) (90:10 *v/v*).

The obtained dispersion was incubated in an orbital shaker at 37 °C and, at predetermined times (0, 1, 2, 4, 7, 12, 16 and 24 h), 3 mL of the medium were taken and equal aliquots of fresh medium were added. Each withdrawn volume was then lyophilized, treated with 1.5 mL of methanol, and centrifuged. Finally, the supernatant was filtered through 0.2 µm cellulose acetate filters and analyzed by HPLC analysis with the conditions described above for Rapa quantification.

#### 2.7.3. Drug Release

A known quantity of Rapa-loaded KP-Nano (Rapa content equal to 0.09 mg) was dispersed in 1 mL of filtered DPBS. The obtained dispersion was placed in a dialysis tube (MWCO of 12–14 kDa) and placed in 30 mL of a DPBS: FBS mixture (90:10 *v/v*). To determine the Rapa diffusion profile across the membrane, a drug dispersion (0.09 mg) was put into the dialysis tube and incubated under the same conditions.

At predetermined times, 3 mL of the external medium were removed and replaced with the same aliquots of fresh medium. Each sample was frozen, freeze-dried, treated with 1.5 mL of methanol, and centrifuged. Finally, the supernatant was filtered through 0.2 µm cellulose acetate filters and analyzed by HPLC analysis with the conditions described above for Rapa quantification.

The content inside the dialysis tube was also lyophilized and subsequently analyzed for HPLC analysis by treating the recovered solid with 400 µL of DMA and then 9.6 mL of methanol.

### 2.8. Evaluation of Storage Stability

The physical and chemical stability of Man/Rapa-loaded KP-Nano as a powder was investigated by following the guidelines of the International Conference on Harmonization (ICH) Q1C and Q1A (R2) [31,32]. In detail, freeze-dried or spray-dried powder samples were stored for 12 months in the dark, either in a freezer at −20 ± 5 °C or in a refrigerator at 5 ± 3 °C. After re-dispersion in bidistilled water, mean size, PDI, and ζ potential were analyzed. The chemical stability of the entrapped drug was determined by HPLC analysis, by following the method reported above.

### 2.9. Biological Characterization

#### 2.9.1. Cell Culture

The line of murine macrophage RAW264.7 cells was chosen for biological experiment and purchased from ATCC (Manassas, VA, USA). Cells were cultured in Dulbecco’s modified Eagle’s medium (DMEM) supplemented with 10% fetal bovine serum (FBS), 2 mM  l-glutamine, 100 U/mL penicillin, and 100 μg/mL streptomycin (all reagents from Euroclone, Milan, Italy). Cells were seeded at 2.5 × 10^4^ cells/cm^2^ and left in an incubator at 37 °C, with 5% CO_2_.

#### 2.9.2. Cell Viability

The viability of RAW264.7 macrophages in the presence of nanoparticles was carried out by a 3-(4,5-dimethylthiazol-2-yl)-5-(3-carboxymethoxyphenyl)-2-(4-sulphophenyl)-2H-tetrazolium (MTS), by using the Cell Titer 96 Aqueous One Solution Cell Proliferation assay kit (from Promega) containing MTS and phenazine ethosulfate. In particular, cells were incubated with an aqueous dispersion (DMEM containing 10% FBS) of Rapa (200 μL per well), free of loaded into KP-Nano or Nano (to obtain drug concentrations of 0.1, 0.05, and 0.01 mg/mL). Cell viability was also evaluated in the presence of empty KP-Nano and Nano, in aqueous dispersion at concentrations corresponding to those of drug-containing systems. All samples before incubation were dispersed in the medium, and the obtained dispersions filtered (0.22 μm cut-off pores). After 24 h of incubation, each well was washed with sterile DPBS after removing the supernatant. Then, the cells in each well were incubated with fresh DMEM, added with 20 μL of an MTS solution, for 2 h at 37 °C. Absorbance values were determined in each well at 490 nm by using a microplate reader (Multiskan Ex, Thermo Labsystems, Vantaa, Finland). Relative cell viability (percentage) was expressed as (Abs490 treated cells/Abs490 control cells) × 100, based on three experiments. Cells incubated with the medium were used as the negative control.

#### 2.9.3. Nanoparticle Uptake

To evaluate the cellular uptake of nanoparticles by macrophages, cells were seeded at 2.5 × 10^4^ cells/cm^2^ and treated the day after with Nano and KP-Nano suspended at 1 μg/mL in the completed medium. After incubation at 37 °C for 2 or 4 h, the cells were washed, fixed with 4 vol% paraformaldehyde in PBS at room temperature, and treated with ActinGreen™ 488 ReadyProbes™ Reagent (Invitrogen™, Carlsbad, CA, USA) to stain the cytoskeleton and with Hoechst 33342 Solution (Thermo Scientific™, Waltham, MA, USA) to stain the nuclei. Images were acquired by a Nikon A-1 confocal microscope and analyzed by the use of NIS-Elements software.

#### 2.9.4. Biological Evaluation of Rapa Activity [ALICE: DA CAMBIARE TESTO]

To evaluate the activity of Rapa, free or loaded into Nano and KP-Nano, macrophages were treated with the same amount of Rapa (free or entrapped into the particles). In particular, RAW264.7 cells were seeded at 2.5 × 10^4^ cells/cm^2^ and treated the day after with Rapa 130 nM, free or loaded into Nano or KP-Nano. Cells were lysed at 24 h after treatments and SDS-PAGE and Western blotting were performed according to standard protocols and as described in [33]. Briefly, 20 μg of proteins obtained from each condition were loaded onto Bolt Bis-Tris gel 4–12% (ThermoFisher Scientific, Waltham, MA, USA) and transferred on nitrocellulose membranes (GE Healthcare; Chicago, IL, USA). Blocking was done in a 5% BSA solution (5% BSA, 20 mM Tris, 140 mM NaCl, 0.1% Tween-20). The following primary antibodies were used for the staining: anti-Phospho-ULK1 (Ser757) (1:500, cat. Number 14202 Cell Signaling Technology, Danvers, MA, USA), anti- ULK1 (D8H5) (1:500, cat. Number 8054 Cell Signaling Technology, Danvers, MA, USA), and anti-α-tubulin (1:1000, cat. number sc398103, Santa Cruz Biotechnology. Dallas, TX, USA). All secondary antibodies were obtained from Thermo Fisher Scientific. The chemiluminescence signal was detected using the ChemiDoc Biorad acquisition instrument and obtained images were analyzed with the Image Lab software (Bio-Rad; Hercules, CA, USA).

## 3. Results and Discussion

In this paper, the design and the production of a novel pharmaceutical formulation for the management of atherosclerosis are described. In particular, with the idea of creating a simple therapeutic system to selectively reduce the growth of atherosclerotic plaques in the vessels of subjects predisposed to atherosclerosis, a powder formulation containing mannitol (Man) and polymeric nanoparticles loaded with rapamycin (Rapa) and targeted to atherogenic macrophages was developed.

### 3.1. Copolymer Synthesis

To achieve this goal, first a biocompatible and biodegradable copolymer was synthesized, starting from materials already used in the pharmaceutical field or already described as potential future materials for the manufacture of pharmaceutical products. Specifically, the copolymer was synthesized by covalently binding appropriate quantities of a fluorescent polyaspartamide, the PHEA-RhB, and a succinate PCL derivate, the PCL-SUCC, to obtain a material (the PHEA-RhB-SUCC-PCL graft copolymer) that can be processed easily up to obtaining nanoparticles, capable of simultaneously incorporating drugs of a hydrophobic nature [27,28].

Each component has a specific function within the produced copolymer. In particular, the PHEA backbone represents the skeleton of the structure to which numerous other functionalities can be conjugated thanks to the presence of a hydroxyl group for each repetitive unit. PCL represents the hydrophobic component that allows the production of water-insoluble nanoparticulate systems, and is therefore able to transport the molecules incorporated in them. The succinic function used to functionalize the PCL beforehand makes the latter more reactive towards the PHEA alcohol groups, allowing for the functionalization reaction to be carried out in milder conditions, while the fluorescent probe RhB allows easy visualization of the resulting system in the technological and in vitro characterization experiments, allowing for an immediate and precise localization, the latter permanently linked to the main polymeric backbone.

**Scheme 1:**
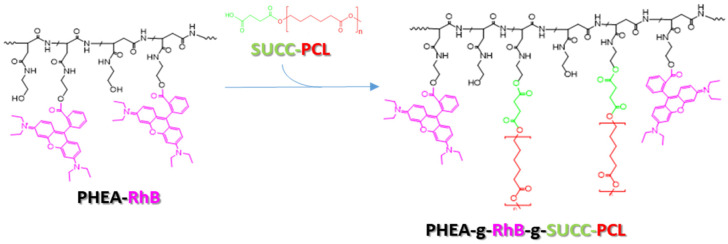
The synthetic route of PHEA-g-RhB-g-SUCC-PCL graft copolymer: PHEA (black), RhB (fuchsia), PCL (red, *n* = 122), succinic acid (green). Reagents and conditions: a-DMF, CDI, DEA, 5 h at 40 °C; 44 h at 40 °C.

In Scheme 1, the synthetic step to obtain the PHEA-RhB-SUCC-PCL graft copolymer is depicted.

The degree of derivatization in PCL (DD_PCL_) was determined from the ^1^H-NMR spectrum, as reported elsewhere, and the result was equal to 2.1 ± 0.3 mol% (see Figure S1 in the Supplementary Materials) [28]. The characterization by size exclusion chromatography (SEC) confirmed the occurrence of the conjugation reaction of PCL-SUCC on PHEA-RhB, being the ${\bar{M}}_{w}$ equal to 136 kDa (${\bar{M}}_{w}/{\bar{M}}_{n}$ = 1.50) (see Figure S2 in the Supplementary Materials).

If compared to the non-labeled polymer, whose synthesis is described elsewhere, there are no significant differences in the quantity of PCL chains grafted to the PHEA backbone, being in both cases between 6 and 7 chains, as well as in the ${\bar{M}}_{w}$. On the other hand, the scarce influence of the presence of covalently linked RhB on the properties of the obtained copolymer had already been observed for derivatives of PHEA with polylactic acid (PLA), recently described [34].

### 3.2. Production of Nanoparticles

Currently, there is some evidence concerning the use of Rapa as an anti-inflammatory drug for the management of atherosclerotic plaques, although its systemic use is limited due to the risks associated with the immunosuppressant effect on circulating and tissue macrophages. For this reason, the Rapa was entrapped into PHEA-g-RhB-g-SUCC-PCL-based nanoparticles actively targeted to atherosclerotic macrophages.

As expected, the obtained PHEA-g-RhB-g-SUCC-PCL graft copolymer was insoluble in aqueous solvents; therefore, it was used as starting material to obtain nanoparticles by the nanoprecipitation technique. In particular, it was dispersed in DMA and then nano-precipitated in water. To obtain nanometer particles with low PDI, preliminary experiments demonstrate that the optimal starting copolymer concentration is 1.5 wt%, at a phase ratio between organic copolymer and aqueous solutions equal to 1/10.

Moreover, to the copolymer dispersion in DMA, the 1-(Palmitoyl)-2-(5-keto-6-octene-dioyl) phosphatidylcholine (KOdiA-PC) was added, at a copolymer:ligand ratio of 60:1. This choice is justified considering that, being a CD36-targeted strong ligand found on oxLDL, its incorporation on the nanoparticle surface could determine the active targeting to atherogenic macrophages. Nanoparticles obtained in the absence or in the presence of KOdiA-PC were named, respectively, Nano and KP-Nano.

Due to the chemical–physical properties of Rapa such as low water solubility, the entrapment of Rapa was successfully carried out during the nanoparticle formation by solubilizing the drug in the organic phase containing both the copolymer and the targeting ligand, respectively, PHEA-g-RhB-g-SUCC-PCL and KOdia-PC.

Due to this strategy, the amount of entrapped drug, evaluated by HPLC analysis and expressed as drug loading (DL%) and entrapment efficiency (EE%), resulted to be equal to 13.7 wt% and 78 wt%, respectively, on the theoretical amount.

After proper purification from free drugs and solvents, the freshly obtained samples were characterized in terms of the mean size, PDI, and ζ potential in bidistilled water.

In effect, the chosen experimental conditions allowed us to obtain Rapa-loaded KP-Nano with a mean size lower than 50 nm and low PDI, being respectively 43.8 nm and 0.25 (see Figure S3 in the Supplementary Materials). Moreover, from data reported in Table 1, it can be seen that the nanoprecipitation process is not significantly influenced by either the entrapment of the targeting ligand and the drug, as the mean size was always lower than 60 nm. Table 1 also shows the ζ potential values, and from these it can be seen that Rapa-KP-Nano shows a negative value of surface charge (−19.2 mV).

### 3.3. Quali-Quantitative Analysis of Kodia-PC

The presence of Kodia-PC among the components used for the preparation of the nanoparticles does not ensure its presence in the final product, nor its presence on the surface of the nanoparticles. The latter is an essential requirement for its function as a directing agent to atherosclerotic macrophages. Therefore, it was necessary to evaluate its presence in nanoparticles, quantify it, and demonstrate that it is found on their surface.

In particular, the quantification of Kodia-PC was carried out by a colorimetric method developed for the quantification of phospholipids in biological samples, which is based on the formation of a complex between phospholipid and ammonium ferrothiocyanate and quantifiable by UV analysis [29]. The analysis made it possible to determine that the quantity of KOdia-PC in the sample is equal to 1.2 wt% with respect to the polymer weight into the KP-Nano sample, which corresponds to approximately 72.3 wt% of the theoretical quantity.

Since Kodia-PC is one of the most potent ligands of CD36, this quantity can be considered acceptable for in vivo administration. The presence of this phospholipid on the surface, essential for the targeting function, was determined by an XPS surface analysis, by analyzing KP-Nano, and Nano as the control sample. In particular, it was found that among the elements exposed on the surface, there is phosphorus, compatible with the phosphate present in the structure of the Kodia-PC, which is absent in the Nano sample. Data, expressed as relative distribution of the carbon, nitrogen, oxygen, and phosphorus species on the nanoparticle surface determined by a curve-fitting procedure of the photoelectron peaks, are reported in Table 2.

### 3.4. Thermal Analysis

**Figure 1:**
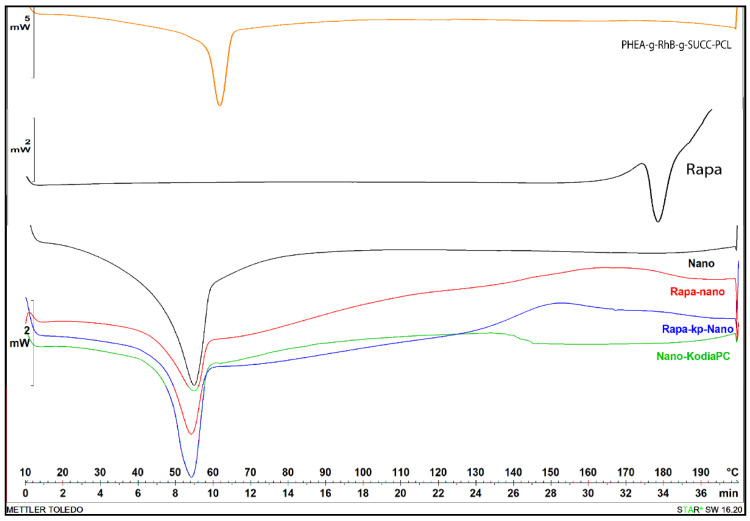
Differential scanning calorimetry (DSC) thermograms of first cooling cycle of Rapa, PHEA-g-RhB-g-SUCC-PCL graft copolymer, empty Nano (black curve), KP-Nano (green curve), Rapa-loaded Nano (red curve) and Rapa-loaded KP-Nano (blue curve).

All obtained nanoparticle samples, as well as raw materials, were characterized by differential scanning calorimetry (DSC) analysis. Transition temperatures and DSC diagrams are reported in Table 3 and Figure 1, respectively.

As can be seen, the DSC diagram of Rapa shows a typical melting endotherm with onset temperature at 178.36 °C indicating its crystalline nature, while the diagram of PHEA-g-RhB-g-SUCC-PCL graft polymer at 69.66 °C, that is similar to PCL melting (the typical endothermic peak of PCL polymer occurred at about 57 °C). The preparation procedure of nanoparticle samples and even the presence of other components such as Kodia-PC seems to slightly affect the melting transition of the polymeric material, being the melting peak for all nanoparticle samples more broadly, and shifted to the lowest temperature (54–55 °C) compared to the raw copolymer. Moreover, in the thermogram of drug-loaded samples, the peak related to the melting point of Rapa is absent, demonstrating that the drug entrapment in the polymeric-based nanoparticle sample showed a different state, such as an amorphous state or disordered-crystalline phase of molecular dispersion or solid solution within the polymer matrix, as other authors have recently reported for Rapa or other molecules [35,36,37]. The thermograms did not reveal any degradation of materials in the range of tested temperature.

### 3.5. Optimization of the Drying Process

The drying has the purpose of obtaining powders, whose extemporaneous dispersion in an aqueous medium allows for the reconstitution of the nanoparticle dispersion with the previously described characteristics.

Although dimensions and surface properties of fresh particles are suitable for intravenous administration, drying by lyophilization produced a solid not easily dispersible in aqueous media, while it would be desirable to have an easily dispersible powder extemporaneously before use. For this reason, mannitol (Man) was selected to be added as the cryoprotectant, which was added to a concentration equal to 1 *w/v*% to the nanoparticle dispersion before the freeze-drying process. The latter was chosen among many, since they are already widely used in the pharmaceutical field as an excipient and as a therapeutically active molecule [38].

As evidenced by values reported in Table 4, the mean size values of all systems in a 1% aqueous solution of Man are almost doubled compared to bidistilled water, albeit always less than 100 nm.

After freeze-drying, it was determined by the mean size and ζ potential measurements that Man presence favors the re-dispersion of nanoparticles in aqueous fluids, while it was difficult in its absence. In particular, after the freeze-drying process, there is a doubling in mean size, which remains below 200 nm, with a slight increase of PDI, being mean size and PDI for Rapa-loaded KP-Nano sample after freeze-drying, respectively, equal to 194.5 nm and 0.32 (see Figure S4 in the Supplementary Materials. This result makes it possible to think of administering such dispersions, for example, intravenously, preparing them before use through a simple dispersion in isotonic solution [14,39].

**Figure 2:**
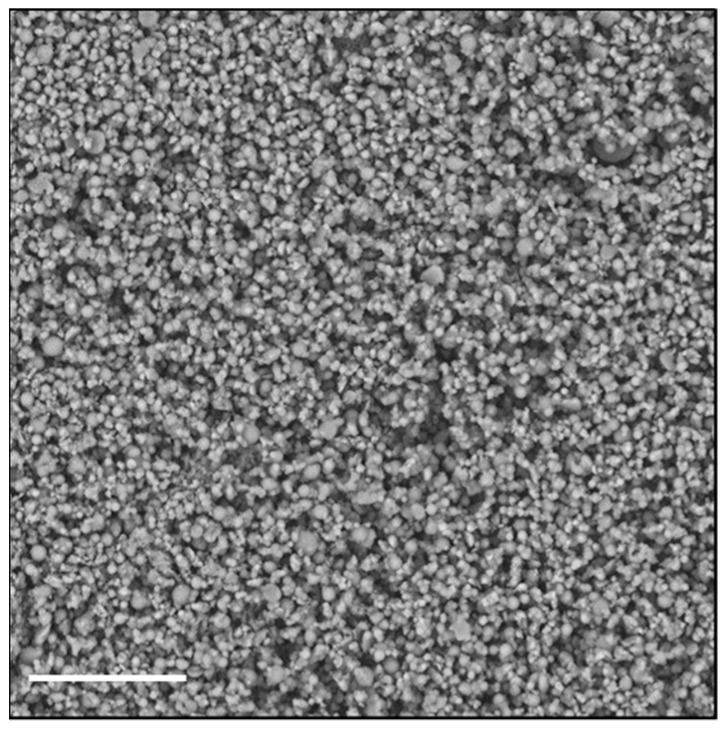
SEM image of microparticles made by mannitol (Man) and Rapa-loaded KP-Nano, obtained by SD. The bar represents 20 μm.

To investigate the possibility of using a different technique to obtain an easily re-dispersible product in aqueous fluids, we chose to dry the nanoparticle dispersion by spray-drying (SD), using Man as a material constituent of the matrix. The advantage of the SD process over lyophilization can be ascribed to the fact that the solid occupies a much smaller volume, and that the powder, being made up of perfectly spherical microparticles of few microns, shows excellent flow properties. Figure 2 shows an SEM image of the particles consisting of Man and Rapa-KP-Nano, obtained by SD.

As can be seen from Table 4, spray-dried Rapa-KP-Nano shows an increase in the average size over those obtained by pre-drying, which remained in a range compatible with intravenous administration. Therefore, the powder formulation, when in contact with an aqueous medium, released empty and Rapa-loaded KP-Nano with a mean size of about 200 nm, comparable with the fresh nanoparticle dispersion.

Moreover, the absence of thermal degradation phenomena on both the drug and the copolymer due to the SD process was evaluated by HPLC and SEC analyses, respectively (data not shown). In particular, the amount of drug incorporated in the Rapa-KP-Nano and Rapa-Nano systems was not significantly changed, being the DL% equal to 13.6 and 13.4 wt%, respectively, after drying by lyophilization and SD. Moreover, the ${\bar{M}}_{w}$ of the copolymer resulted to be equal to 128.5 kDa (${\bar{M}}_{w}/{\bar{M}}_{n}$ = 1.56); therefore, it can be superimposed on the value determined once synthesized. This demonstrates that the experimental conditions chosen to carry out the SD process in terms of nebulizer temperature, flow, and spray rate of the feed liquid are suitable. These do not cause any degradation of both the PHEA-RhB-SUCC-PCL copolymer and the drug, which is known to be sensitive to high-temperature values and therefore could degrade in the nebulizer nozzle.

### 3.6. Drug Stability and Release from KP-Nano

Having a macrolide structure, rapamycin undergoes chemical instability in physiological fluids [40,41]. Therefore, the entrapment into a nanoparticle-like structure could increase the drug solubility in these media, protect the drug from degradation phenomena, and allow a controlled release of the entrapped drug, maintaining a proper drug concentration in biological fluids as well as in the accumulation site.

**Figure 3:**
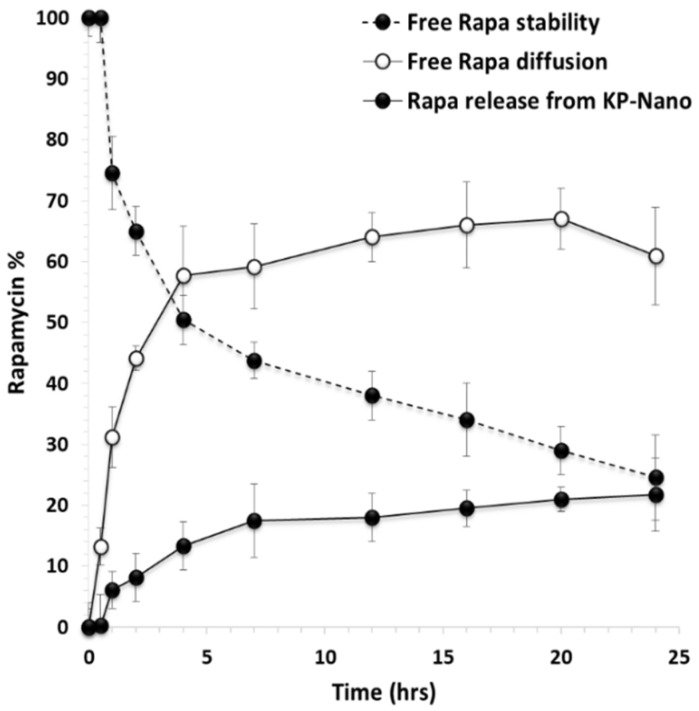
Free Rapa stability (- -●- -), free Rapa diffusion (-o-) and Rapa release profile (-●-) from KP-Nano across the dialysis membrane in DPBS/fetal bovine serum (FBS) (pH 7.4). Data represent mean ± S.D., (*n* = 3).

To evaluate the effect of entrapment into KP-Nano on the stability of the drug, a stability study was carried out in DPBS: fetal bovine serum (FBS) 9:1 *v/v* at pH 7.4, to mimic the physiological conditions. The presence of FBS increases drug solubility as well as the degradation processes [42,43,44]. In particular, a drug-loaded KP-Nano dispersion was incubated and put into a dialysis tube, which was immersed into FBS-enriched DPBS. To compare the drug’s stability when it is incubated in the same medium in free form, the same experiment was carried out incubating a free drug dispersion in the same medium (at the same concentration of that entrapped into the KP-Nano). At chosen interval times, the amount of intact drug was quantified by HPLC and reported in Figure 3.

It can be noted that, concerning the free Rapa stability, the drug concentration in the incubation medium decreases over time, reducing up to 24.6 wt% after 24 h of incubation. In the release study of the drug from the KP-Nano, the amount of intact drug released in the medium after 24 h of incubation is equal to 21.8 wt% with respect to the total entrapped drug, while that unreleased and still found in the nanoparticles the 50.2 wt%. Therefore, the entrapment of Rapa into KP-Nano produces 47.4 wt% of protection of the entrapped drug from degradation after 24 h of incubation, being the amount of total intact drug (released + trapped) in the KP-Nano equal to 72 wt% of the total. This result confirms the potential advantages of administering an unstable drug such as Rapa in biological fluids within a colloidal vector such as polymeric nanoparticles, reducing its degradation, keeping a greater amount of drug intact in the biological fluid in which it is dispersed, and consequently favoring the achievement of the same in the target site in an active form.

From what has been reported so far, the followed strategy made it possible to obtain nanoparticles containing Rapa, which were embedded into a Man-based powder. The latter, once that the administration of the aforementioned particles is necessary, is extemporaneously dispersed in an aqueous medium, and can dissolve to reconstitute a dispersion of nanoparticles that are capable in turn of protecting the drug and releasing it in a controlled manner.

### 3.7. Long Term Storage

Once it was demonstrated that the KP-Nano systems protect the entrapped Rapa from hydrolysis and release it in a prolonged way in the medium, it was essential to determine the stability of the formulation after storage in the form of powder under different conditions.

In particular, the stability of either freeze-dried or spray-dried Man/Rapa-loaded KP-Nano was determined after storage of the powder in terms of size, PDI, and ζ potential, according to International Conference on Harmonization (ICH) guidelines Q1C and Q1A (R2) [31,32]. In detail, a proper amount of dried powder was stored for 12 months either in a freezer at −20 ± 5 °C or in a refrigerator at 5 ± 3 °C. After this time, each sample was dispersed in bidistilled water, and the physical appearance and ease of reconstitution were both evaluated. Moreover, the obtained dispersion was analyzed in terms of mean size, PDI, and ζ potential. Data reported in Table 5 suggest that the dried formulations are stable under storage in the reported conditions, being mean size and ζ potential values after re-dispersion comparable to those measured after drying (reported in Table 2). IN addition, the chemical stability of Rapa entrapped into NPs was confirmed by HPLC analysis after storage (data not shown).

### 3.8. Cellular Viability, Internalization and Validation of Rapa-Nano Activity

**Figure 4:**
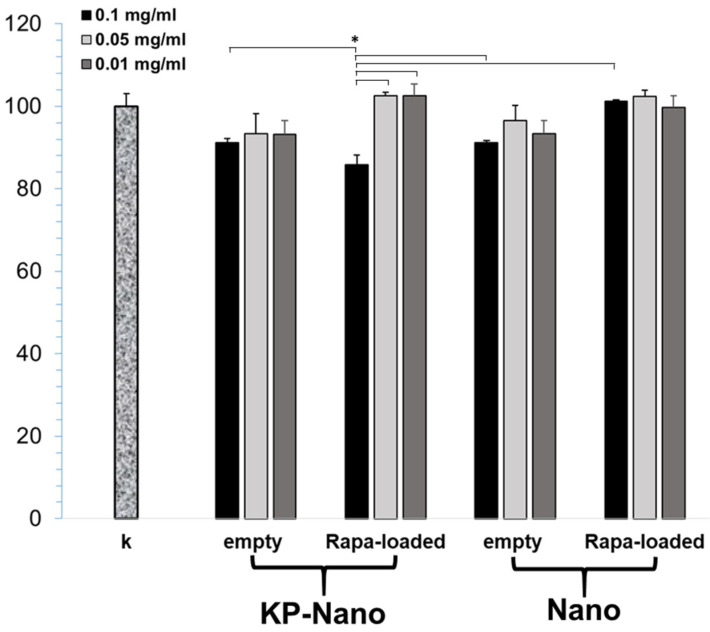
MTT assay of RAW264.7 cells after 24 h of incubation with Rapa loaded into KP-Nano or Nano, and empty KP-Nano or Nano. Cell viability expressed as % respect to untreated cells. (* *p* < 0.001).

To validate the carriers, we first excluded any toxicity on the macrophage RAW264.7 cell line. As shown in Figure 4, three different concentrations of nanoparticles were tested and, in most cases, the reduction in cell viability was not significant compared to the control 24 h after treatment. A slight reduction, less than 20%, has been observed in cells treated with Rapa-KP-Nano, and this reduction was found to be significantly greater than that obtained with the same particles carrying lower drug concentrations. Moreover, it also resulted significantly concerning the untargeted particles carrying the same amount of drug (0.1 mg/mL), and concerning the empty systems (at the concentration needed to incorporate the same amount of drug). This effect on the cell viability of the Rapa-loaded KP-Nano samples could be due to the effect of the drug, which is more internalized due to the entrapment into the targeted nanoparticles.

**Figure 5:**
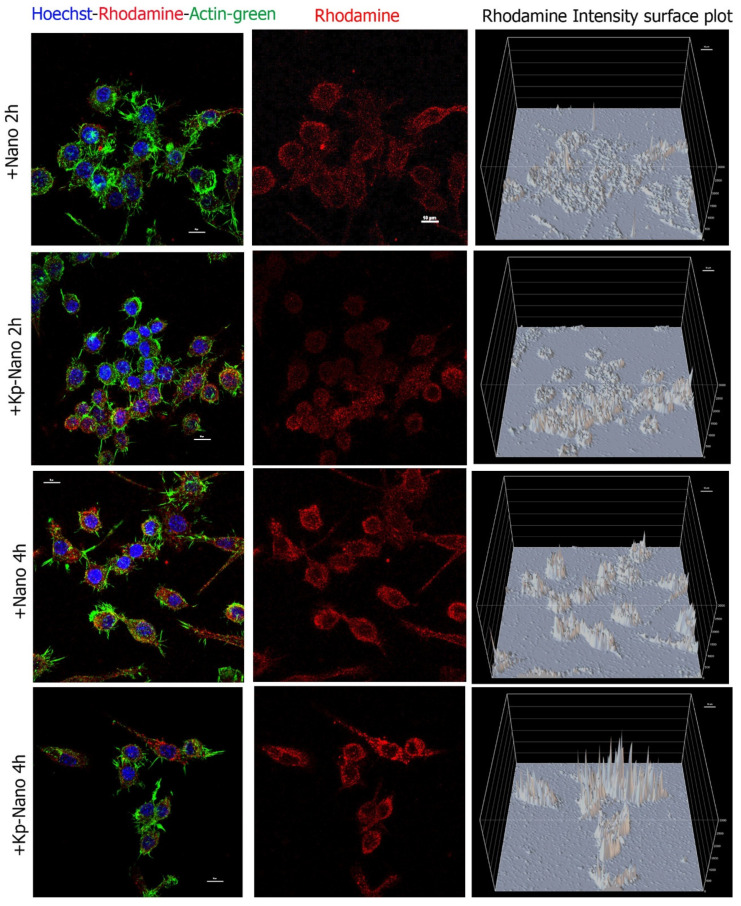
Cellular internalization. Confocal analyses of RAW264.7 cells treated for 2 h and 4 h with Nano or KP-Nano (both in red) and stained with ActinGreen™ 488 for the cytoskeleton (in green) and with Hoechst 33342 (in blue) for the nuclei. The left and middle panels show the maximum intensity projection of five focal planes, with the merge of the three signals on the left and only the rhodamine in the center. The scale bar is 10μm. The right panels show the intensity of rhodamine fluorescence for pixel; in the graphs, the fluorescence intensity is reported as an arbitrary unit of fluorescence (auf) and the scale of the grid is 500 auf.

Internalization assay was performed to prove the uptake of Nano and KP-Nano by cells and to test the targeting effect of the KP phospholipid on these cells. To do this, macrophage RAW264.7 cells have been treated with Nano and KP-Nano suspended in growth medium at 1 μg/mL and analyzed by confocal microscopy after 2 h and 4 h (Figure 5). As expected by the nature of macrophages, both systems are already internalized by cells after two hours; however, the amount of KP-Nano appears to be higher, as evidenced by the intensity of fluorescence, data that is most evident after 4 h of treatment.

**Figure 6:**
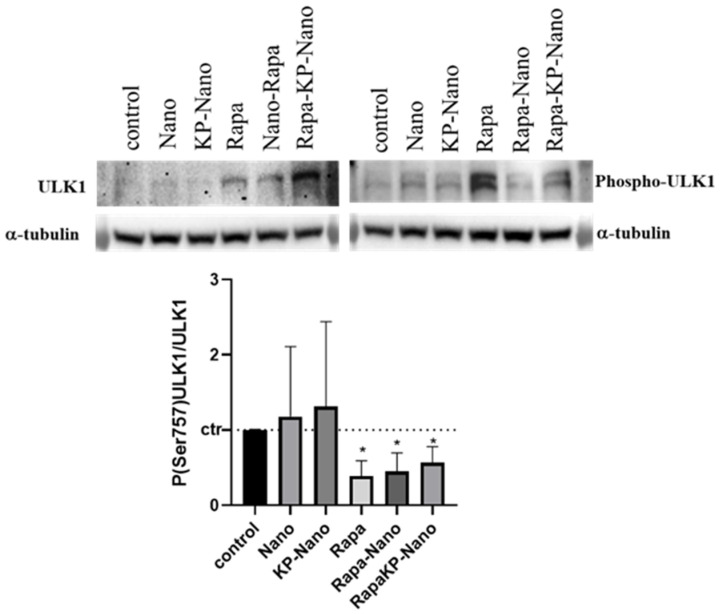
Evaluation of Nano-Rapa activity. Upper panel: Representative image of Western blots for ULK1, Phospo-ULK in Ser 757 and α-tubulin. Protein extracted from cells untreated or treated with the different compound for 24h. Bottom panel: Densitometric analyses of the biological replicates. The graphs show the ratio between the optical densities (ODs) of P(Ser757)ULK1 and ULK1, both previously normalized for the ODs of the respective α-tubulin. The values are represented vs. untreated cells. * *p* value < 0.05.

Finally, to investigate the activity of Rapa entrapment into nanoparticle systems, we investigated the effects of the different compounds on the protein kinase mTOR, a direct target of Rapa. In physiological conditions and in the presence of nutrients, mTOR phosphorylates the Ser758 (Ser757 in mouse) of the autophagy activating kinase ULK1, inhibiting its activity [45]. Rapa treatment inhibits the kinase activity of mTOR with a subsequent reduction in ULK1 phosphorylation and autophagy activation [46]. To compare the activity of the nanosystems with that of Rapa free, we treated RAW264.7 cells with the same concentration of drugs free or entrapped in nano or KP-Nano and investigated the level of phospho-Ser757-ULK1, 24 h after treatment. Representative images of Western blotting and densitometric analyses are reported in Figure 6. As shown, Rapa entrapped into KP-Nano induced a significant reduction of Phospo-Ser757-ULK1 protein, in a manner comparable to free Rapa. The same effect is evidenced also by Rapa loaded into Nano, while any reduction was revealed after the treatment with empty nano. These data confirmed that the entrapped drug maintains its activity on cells in culture.

## 4. Conclusions

In this paper, a novel pharmaceutical formulation potentially useful for the management of atherosclerosis was developed. In detail, an advanced pharmaceutical formulation for Rapa administration was designed and optimized to be extemporaneously dispersed in a physiological medium giving rise to a colloidal dispersion of nanoparticles ready to be administered in vivo. To do this, a proper polymeric material was synthesized and polymeric nanoparticles were produced by nanoprecipitation. During the process, KOdia-PC, an oxidized phospholipid with the highest affinity for the macrophage CD36 receptor, was also entrapped to realize a carrier targeted to the atherogenic macrophages. In effect, it was demonstrated that the targeted ligand was mainly on the nanoparticle surface. The obtained particles were smaller than 100 nm, and trapped a large amount of the drug in amorphous form. Moreover, the lyophilization in the presence of Man allows for reconstituting the formulation with characteristics practically comparable to the initial ones and suitable for parenteral administration. The powder formulation was also stable to storage and, once redispersed in a physiological medium, was able to protect the entrapped drug from degradation and release it in a controlled manner.

Biological studies on macrophages have shown that nanoparticles do not induce toxicity in cells even at high concentrations, while uptake assay confirmed nanoparticles internalization, even if in vivo studies are required to confirm the efficiency of CD63 targeting. Moreover, the drug carried into the nanoparticles, targeted and not, has efficacy in inhibiting the mTOR pathway in a manner comparable to the free drug. This result is important as it demonstrates that Rapa incorporated into either KP-Nano or Nano samples is more stable than the free drug when incubated in fluids mimicking physiological ones. The fact that the free drug degrades under these conditions is widely reported and demonstrated in the literature [40,41]. Therefore, we can suppose that since there is a certain Rapa amount entrapped into the nanoparticles still intact in the medium after 24 h of incubation, the drug effect due to entrapment into nanoparticles could be more lasting over time, even if further studies are required to prove this. Furthermore, it is widely known that, when administered as an immunosuppressant in tablets, the oral bioavailability of Rapa is currently very low (approximately 15% of the administered dose), which could be attributed mainly to its sensitivity to gastric acid, incomplete GI absorption, and first-pass hepatic metabolism [7,8]. In this case, we can assume that the parenteral administration of the drug entrapped into KP-Nano carriers may require a lower dosage of the drug than that necessary for oral administration, and also because the fraction of free drug absorbed by the GI tract may not preferentially accumulate in the site target concerning to targeted nanoparticles.

## Figures and Tables

**Table 1:** Mean size, polydispersity index (PDI) and ζ potential values of empty and rapamycin (Rapa)-loaded untargeted particles (Nano) and nanoparticle surface (KP-Nano) in bidistilled water. Data represents the media of three measurements.

Sample	Mean Size (nm ± S.D.)	PDI ± S.D.	ζ Potential (mV ± S.D.)
Nano	56.6 ± 22.4	0.25 ± 0.03	−17.2 ± 2.8
Rapa-Nano	57.3 ± 23.2	0.24 ± 0.07	−15.2 ± 3.0
KP-Nano	50.9 ± 18.8	0.26 ± 0.10	−10.3 ± 3.5
Rapa-KP-Nano	43.8 ± 27.4	0.25 ± 0.05	−19.2 ± 1.6

**Table 2:** XPS surface chemical composition of KP-Nano and Nano ^a^.

Sample	C 1s	O 1s	N 1s	P 2p
**KP-Nano**	68.45	26.21	5.20	0.14
**Nano**	69.70	26.40	3.90	---

^a^ Relative distribution of the carbon, nitrogen, oxygen, and phosphorus species on the nanoparticle surface determined by a curve-fitting procedure of the photoelectron peaks.

**Table 3:** Transition temperature values of nanoparticle samples and raw materials.

Samples		T_M_/°C
**Raw materials**	Rapa	178.36
	PHEA-g-RhB-g-SUCC-PCL	69.66
**Nano**		54.73
**Rapa-Nano**		54.71
**Rapa-KP-Nano**		54.09
**KP-Nano**		54.08

**Table 4:** Mean size, PDI and ζ potential values of empty and Rapa-loaded Nano and KP-Nano in bidistilled water, after freeze-drying or spray-drying (SD) in the presence of man. Data represent the media of three measurements.

Sample	Mean Size	PDI ± S.D.	ζ Potential
	(nm ± S.D.)		(mV ± S.D.)
**Pre-drying**			
Nano	86.3 ± 16.2	0.30 ± 0.06	−19.1 ± 2.5
Rapa-Nano	97.6 ± 24.7	0.32 ± 0.12	−18.7 ± 3.2
KP-Nano	92.6 ± 16.7	0.37 ± 0.08	−10.6 ± 2.1
Rapa-KP-Nano	98.1 ± 15.0	0.31± 0.05	−21.2 ± 3.3
**Post-freeze drying**			
Nano	170.7 ± 12.2	0.33 ± 0.07	−10.4 ± 0.8
Rapa-Nano	198.6 ±17.5	0.23 ± 0.11	−20.4 ± 6.1
KP-Nano	188.1 ± 20.7	0.37 ± 0.04	−10.9 ± 1.4
Rapa-KP-Nano	194.5 ± 21.3	0.32 ± 0.05	−21.7 ± 4.9
**Post-spray drying**			
Nano	139.3 ± 24.5	0.48 ± 0.12	−12.6 ± 0.9
Rapa-Nano	161.1 ± 20.8	0.35 ± 0.09	−8.1 ± 4.9
KP-Nano	178.0 ± 20.1	0.36 ± 0.06	−14.6 ± 0.9
Rapa-KP-Nano	213.8 ± 19.6	0.40 ± 0.07	−11.4 ± 6.0

**Table 5:** Mean size, PDI and ζ potential values in bidistilled water of Man/Rapa-loaded KP-Nano, freshly freeze-dried or spray-dried (**t**_0_), and upon storage for 12 months at −20 °C (t_12months_, −20 °C) or at 5 °C (t_12months_, 5 °C).

**Mean Size (nm ± S.D.)** **(PDI ± S.D.)**			
	**t_0_**	**t_12months_, −20 °C**	**t_12months_, 5 °C**
***After freeze-drying***	194.5 ± 21.3 (0.32 ± 0.05)	201.5 ± 28.5 (0.34 ± 0.11)	220.3 ± 20.2 (0.37 ± 0.08)
***After spray-drying***	213.8 ± 19.6 (0.40± 0.07)	209.7 ± 23.6 (0.40 ± 0.08)	217.7 ± 22.5 (0.41 ± 0.09)
**ζ potential (mV) (±S.D.)**			
	**t_0_**	**t_12months_, −20 °C**	**t_12months_, 5 °C**
***After freeze-drying***	−21.7 ± 2.3	−18.6 ± 4.4	−17.6 ± 4.1
***After spray-drying***	−11.4 ± 6.0	−15.7 ± 6.9	−16.0 ± 3.0

## Data Availability

Not applicable.

## Supplementary Materials

The following are available online at https://www.mdpi.com/article/10.3390/pharmaceutics13040503/s1, Figure S1: ^1^H NMR spectra of PHEA-g-RhB, PCL-SUCC, and PHEA-g-RhB-g-SUCC-PCL copolymers in DMF-d7, Figure S2: Size Exclusion Chromatography (SEC) chromatograms for PHEA-RhB (grey line) and PHEA-g-RhB-g-SUCC-PCL graft copolymers in DMF + LiBr 0.01M, Figure S3: Distribution size (intensity %) of sample Rapa-loaded KP-Nano, determined by DLS, Figure S4: Distribution size (intensity %) of sample Rapa-loaded KP-Nano after freeze drying, determined by DLS.
