# Supplementary material for: Rapamycin-Loaded Polymeric Nanoparticles as an Advanced Formulation for Macrophage Targeting in Atherosclerosis
Source: Pharmaceutics. 2021 Apr 7;13(4):503. doi: 10.3390/pharmaceutics13040503 (PMC8067637; doi:10.3390/pharmaceutics13040503)
Supplement: Supplementary file 1 [file pharmaceutics-13-00503-s001.pdf]

pharmaceutics

# Supplementary Materials: Rapamycin-Loaded Polymeric Nanoparticles as an Advanced Formulation for Macrophage Targeting in Atherosclerosis

**Emanuela Fabiola Craparo, Marta Cabibbo, Alice Conigliaro, Maria Magdalena Barreca, Teresa Musumeci, Gaetano Giammona, Gennara Cavallaro**

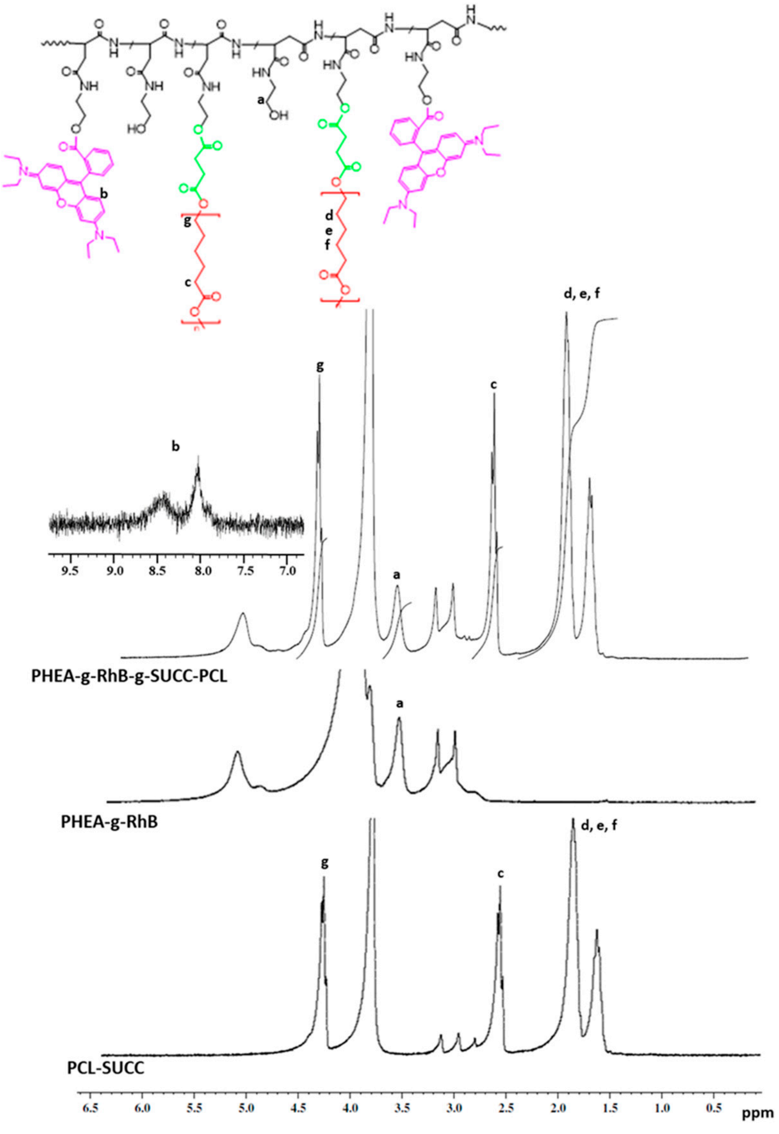


| | Integral values | | |
|---|---|---|---|
| Peak in the spectrum | b | a | d,e,f, |
| Attribution | $10H_{RhB}$ Ar-H | $2H_{PHEA}$ $-C(O)CHCH_2C(O)NH-$ | $6H_{PCL}$ $-[O(O)CCH_2(CH_2)_3CH_2]_{122}-$ |
| **PHEA-g-RhB** | 0.12 ± 0.05 | 1 | ---- |
| **PHEA-g-RhB-g-SUCC-PCL** | 0.12 ± 0.05 | 1 | 7.7 ± 0.3 |

**Figure S1.** $^1$H NMR spectra of PHEA-g-RhB, PCL-SUCC, and PHEA-g-RhB-g-SUCC-PCL copolymers in DMF-d7.

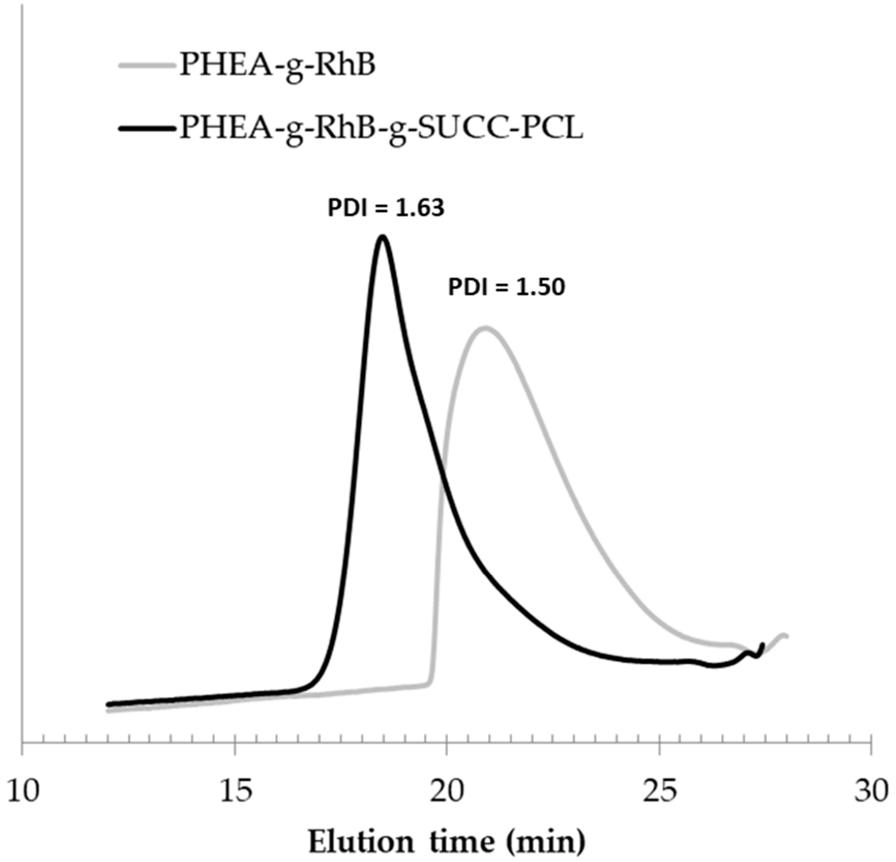


**Figure S2.** Size Exclusion Chromatography (SEC) chromatograms for PHEA-RhB (grey line) and PHEA-g-RhB-g-SUCC-PCL graft copolymers in DMF + LiBr 0.01 M.

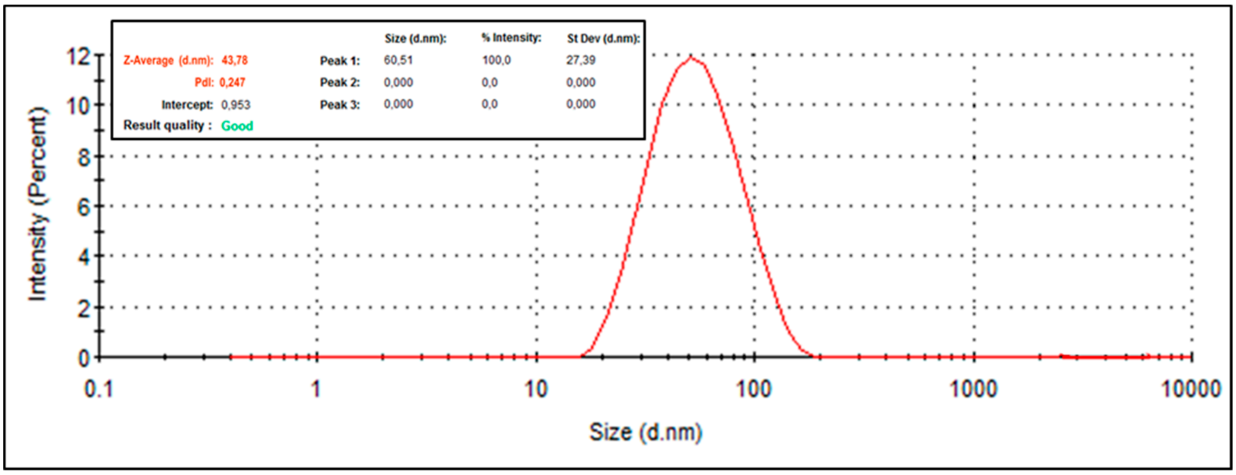


**Figure S3.** Distribution size (intensity%) of sample Rapa-loaded KP-Nano, determined by DLS.

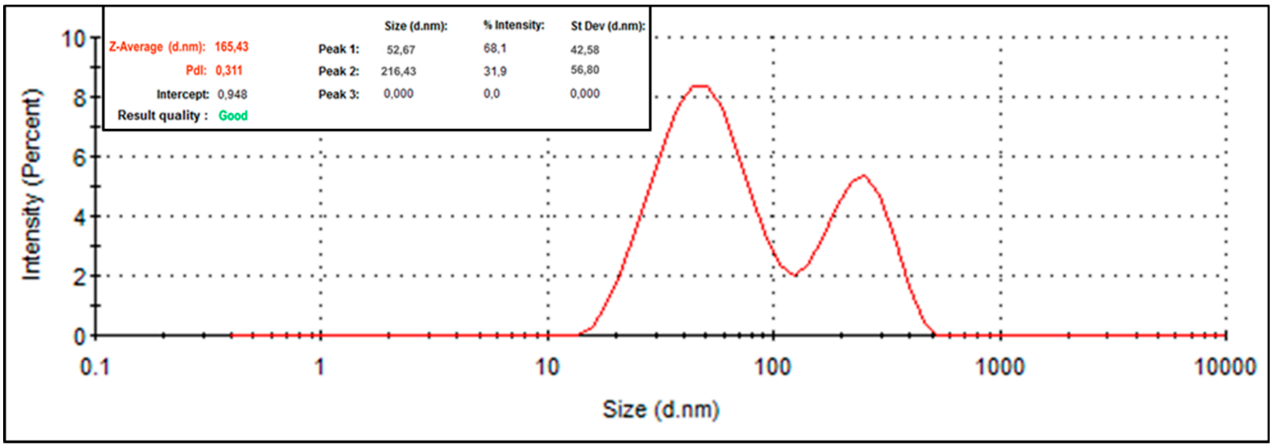


**Figure S4.** Distribution size (intensity%) of sample Rapa-loaded KP-Nano after freeze drying, determined by DLS.
